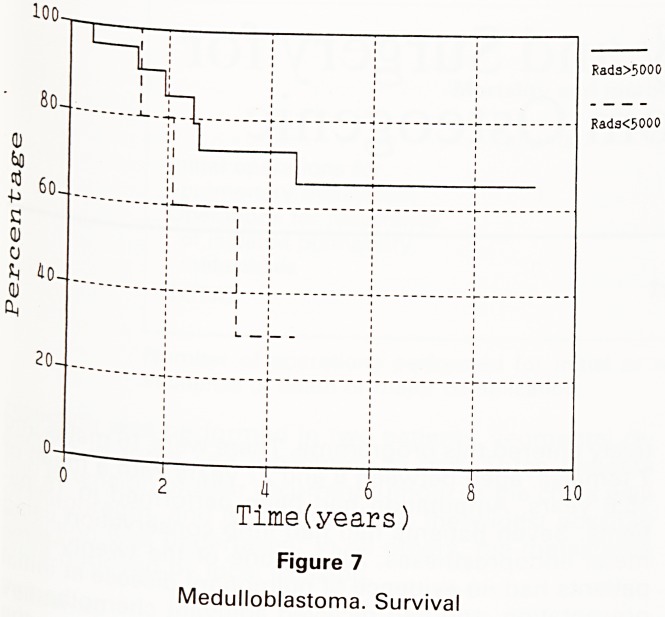# Medulloblastoma in the South West

**Published:** 1988

**Authors:** Nick Foreman

**Affiliations:** Bristol Children's Hospital


### Childhood Medulloblastoma in the South-West 1976–1986

**Nick Foreman**
Bristol Children's Hospital

### INTRODUCTION

Tumours of the central nervous system, taken as a group, are the commonest form of solid tumour in childhood. The contribution of chemotherapy to improved prognosis, so important in other childhood malignancies, has been limited, in part because of the blood-brain barrier. Nevertheless improvements in surgery, radiotherapy and in supportive care have made a demonstrable impact on prognosis in some, but not all, types of CNS tumours.

A retrospective study of children under 16 years who presented with primary intracranial tumours in the last decade in the South-West Region was undertaken. As children with brain tumurs presenting in Bath are traditionally treated in Bristol, these were included. Children received surgery in 2 neurosurgical centres, 141 patients were identified of whom 49 were treated in Plymouth and 92 in Bristol. Retinoblastomas and secondary tumours were excluded. Information was obtained from neurosurgical, radiotherapy and paediatric notes concerning tumour type, treatment given, and outcome. Insufficient information was available about eight identified patients, none of whom appears to have had medulloblastoma, and these were excluded from further analysis. The object of the study was to identify factors influencing outcome. Special attention was paid to those children with medulloblastoma.

The average age was 6,7 years for the whole series (range 6 weeks to 15 years). In comparison to national series astrocytomas are over-represented (46.2%) and ependymomas (8.5%) under-represented. However the American Surveillance Epidemiological Evaluation Results registry (S.E.E.R.) has astrocytomas at 44% and ependymomas at 9%, similar proportions to those in the South-West.

Medulloblastomas formed 25% of the S.E.E.R. registry of primary intracranial neoplasms, [Ref 1] and form the largest single group in this series with 39 patients. It is an interesting group in that the outcome has improved compared with previous decades, and yet a substantial mortality and morbidity remains. This contrasts with brain-stem gliomas where the outlook has remained almost uniformly poor and cerebellar astrocytomas where the outlook is almost uniformly good. Medulloblastomas are malignant tumours arising in the posterior fossa, with the potential for local invasion of the cerebellum and for distant metastases throughout the CNS. There is also a potential for metastases outside the CNS, especially if a ventriculo-peritoneal shunt has been inserted. Medulloblastoma often presents with hydrocephalus due to blockage of CSF pathways, and ataxia.

**Table 1**

**Tumours occurred in the following proportions**

| | |
|---|---|
| Medulloblastomas | 27.0% |
| Brain-stem Gliomas | 11.3% |
| Cerebellar astrocytomas | 17.0% |
| Supratentorial gliomas | 17.0% |
| Optic gliomas | 7.0% |
| Ependymomas | 8.5% |
| Pineal tumours | 5.6% |
| Craniopharyngiomas | 3.5% |
| Meningiomas | 2.1% |
| Choroid plexus tumours | 2.1% |
| Others | 3.5% |

### The Medulloblastoma group

Of the 39 patients treated in the SWR with medulloblastoma, 28 were treated in Bristol and 11 in Plymouth. All had the diagnosis confirmed histologically. There was a heavy male predominance (31 to 8). This predominance was especially marked in the Plymouth group, where only 1 out of 11 patients was female. The mean age was 6 years 11 months (range 2 months to 13 years 9 months). The mean age of the children presenting in Plymouth was higher at 8 years 1 month compared to 6 years 2 months in Bristol.

Of the Bristol patients 14 were from Avon and 14 were regional referrals (Gloucestershire 7, Wiltshire 3, Somerset 4). Plymouth patients were drawn from Cornwall (4) and Devon (7).

### Treatment

The initial treatment was surgical, often with insertion of a shunt before debulking of the tumour.

Of the 28 children treated in Bristol 21 (75%) had total or subtotal resections (greater than 80%), 3 (11%) had partial resections (less than 50% removed) and 4 were unresectable (14%). Of the 11 patients treated in Plymouth 9 (82%) had subtotal resections, 1 (9%) had a partial resection and 1 child died operatively during an initial shunt procedure.

Most children went on to receive radiotherapy. Seven children treated in Bristol did not do so because of untreatable extensive disease (1 patient), failure of post-operative recovery (4 patients) or devastating post-operative infection (2 patients). Two patients in Plymouth did not receive radiotherapy, due to failure of post-operative recovery (1 patient) and operative death (1 patient).

The dosage of radiotherapy in the Bristol patients was standardised and supervised by 1 consultant radiotherapist. This consisted of 50–55 Gy to the posterior fossa, 35 to the skull, and 35 to the spine [as in figure 1]. In fact of this group 1 received 50, 1 received 53 and the rest

**Figure 1**

Dosage of Radiotherapy received 55 Gy to the posterior fossa. In Plymouth, the dose of radiation was less in 6 out of 7 cases where the dosage has been determined. Dosages were as follows:

| *Posterior Fossa* | *Skull* | *Neck* | *Spine* |
|---|---|---|---|
| 45 | 45 | 35 | 31 |
| 45 | 25 | | 25 |
| 35 | | 33 | 26 |
| 30 | | | 27 |
| 30 | | | |
| 40 | | | |
| 55 | 35 | | 35 |

The last patient was also the most recently treated and received his treatment on the most recent SIOP trial. His radiotherapy was the same as that routinely administered throughout the last decade in Bristol. Patients in both centres received chemotherapy with vincristine during radiotherapy.

In the two centres combined there were 31 patients who could have received further chemotherapy, in addition to the vincristine given during radiotherapy. Only 8 did so, 6 of the Bristol patients and 2 of the Plymouth patients. Of the 22 patients in Bristol who could have received chemotherapy, 6 were randomised in either the UKCCSG or the International Society of Paediatric Oncology (SIOP) trials to have the non-chemotherapy arm. In 1 case chemotherapy was refused by the parents. This leaves 9 patients, clustering in the 1981–1984 period, in which there was a clinical decision not to offer chemotherapy.

4 of the 6 patients in Bristol receiving chemotherapy did so on the first SIOP trial. This consisted of 9 doses of vincristine followed by 8 courses of vincristine and CCNU given at 4 weekly intervals. This treatment was not easily tolerated and 2 patients discontinued chemotherapy at an early stage. One patient received CCNU and Procarbazine at 6 to 8 week intervals on the UKCCSG trial. A further patient received a combination of VM26, D.T.I.C. and Procarbazine (a pilot protocol).

The chemotherapy details of 1 patient who was treated neurosurgically in Plymouth are not available as he received it at the referring hospital. The other patient who received chemotherapy in Plymouth did so on the current SIOP trial. This consisted of initial Procarbazine and high dose Methotrexate before radiation ("sandwich" therapy), followed by cycles of vincristine and CCNU.

In all 12 of the 39 patients (31%) were treated on national or international trials.

### Results

The 5 year actuarial survival for all 141 brain tumour patients traced in the SWR was 56% (figure 2).

Of the 39 children with medulloblastoma, 19 are alive with no evidence of disease, 2 are alive with evidence of disease and 18 are dead, 7 patients are alive without evidence of disease more than 5 years after diagnosis. The 5 year actuarial survival is 45% (figure 3) and 5 year event free survival 40%.

Most of the deaths occurred shortly after diagnosis, 7 within one month and 12 within a year. Six of the 7 very early deaths were operative or perioperative; 1 died operatively, 1 had a post-operative arrest, 3 failed to regain conciousness and 1 died as a result of meningitis. In addition one death at seven months was attributable to poor post-operative recovery. Two children were left with very severe handicaps post-operatively (1 after encephalitis). One of these 2 (who was blind with spastic quadriplegia) died some 2 years after diagnosis but it is not certain whether there was any residual disease as no post-mortem was performed.

**Figure 2**

Survival Time—Brain Tumours 141 patients. 1976–86

Two children died of unresectable disease shortly after presentation. Eight children had recurrent disease, usually at multiple sites especially in the spinal column. One child, who had a frontal lobectomy for recurrent disease there, subsequently developed spinal secondaries.

Girls did better than boys, with 71% 5 year actuarial survival versus 43% survival (figure 4). Indeed of the 7 survivors disease free after 5 years, 4 are girls in spite of the fact that they make up less than a quarter of the sample.

Two out of 8 girls died; 1 of unresectable disease and the other of a post-operative arrest. No girl whose tumur was resectable has had recurrent disease.

The Bristol patients did better than the Plymouth patients with a 5 year survival of 52% versus 29% (figure 5) and a 5 year event free survival of 43% versus 23%.

Long-term morbidity must be considered in any review of intracranial tumours. Of the 13 Bristol patients who survived more than 2 years from diagnosis 10 (77%) received growth hormone therapy. Three were treated with thyroxine and 1 required testosterone and oestrogen to induce puberty. No patient in Plymouth has received growth hormone. This may be a true effect due

**Figure 3**

Survival Time—Medulloblastomas 39 patients 1976–86

**Figure 4**

Medulloblastoma. Survival by sex

**Figure 6**

Medulloblastoma. Survival excluding girls

**Figure 5**

Medulloblastoma. Survival: 1976–86

to the lower dosage of radiotherapy but these patients have not been formally assessed for growth hormone deficiency. Even with growth hormone there is likely to be a shorter final height due to the spinal radiation. The magnitude of this effect needs studying once all children, who survive and have hormone replacement therapy if indicated, have reached their final height.

Severe handicap was present in 1 patient treated in Bristol who subsequently died of leukaemia, 1 patient has moderate ataxia and a persisting 6th nerve palsy. 1 patient in Plymouth was blind and had a spastic quadreplegia, dying 2 years after initial presentation. Out of 10 survivors of medulloblastoma assessed by Elliot et al in Bristol, 6 had gaze evoked nystagmus and 4 had reduced visual acuity [Ref 2].

**DISCUSSION**

This group of 39 patients was defined by presentation to the 2 neurosurgical centres in the SWR. It may therefore exclude patients who died before surgical referral. Male predominance is noted in most series, although the sex ratio is not usually as marked as in this group (ratios vary from 3:1 to 1,1:1) [Ref 3,4,5]. The true incidence in the SWR is difficult to estimate, given the difficulty in drawing a referral boundary in Wiltshire, but appears to be 4 presentations per year from a childhood population of approximatley 420,000 under 16, giving a crude incidence of 1 in 100,000. This is higher than the incidence ascertained in the Manchester Children's Tumour Registry of 1 in 190,000 [Ref 6].

The survival of children with medulloblastoma has shown considerable improvement over time. Of 10 children treated in Bristol in the period 1969 to 1975, none survived more than 2½ years [Ref 7]. In an historical review by Chatty and Earle in 1971, of their 67 patients aged under 15 years, only 3 lived to 5 years and 1 lived to 10 years [Ref 5]. The most recent large European series (SIOP, reported in Sept, 1986) has a 5 year survival of 53% and a disease free survival of 48%. This is better than that achieved in the SWR as a whole but is very similar to the 5 year survival of 52% and 5 year event free survival of 43% amongst the Bristol patients.

With the small numbers concerned, the difference in survival between Plymouth and Bristol might be due to chance. The difference between the 2 groups is not explained by the larger proportion of girls in the Bristol group. If only boys are compared then the boys treated in Bristol have a 46% actuarial 5 year survival against a 19% survival in Plymouth (figure 6). Given that only 6 children were treated with chemotherapy in Bristol (and 2 of them discontinued very early) and 2 in Plymouth, there is little to suggest that a more aggressive attitude to chemotherapy is responsible for the difference.

A clearly identifiable difference between the 2 centres in the period studied is the radiation dosage given to the posterior fossa in the past decade. If those who received more than 50 Gy are compared to those who received less than 50 Gy there is a 4 year survival of 65% as compared to 30% (figure 7). Given the variation in the regimes used in Plymouth in the past decade it is difficult to be certain that the biological dosage delivered was usually less than in Bristol, although this seems likely. McIntosh in a review of the improved prognosis in medulloblastoma felt that much of the improvement was due to changes in radiotherapy regimens including the introduction of a higher dose. In his series of patients treated at Great Ormond Street, the only survivors were

**Figure 7**

Medulloblastoma. Survival the 18 patients who had surgery and a dose to the posterior fossa of more than 50 Gy [Ref 7]. In a large British study survival rates were significantly higher in those who received more than 45 Gy to the posterior fossa [Ref 8]. It is of interest that the larger dose has now been used in Plymouth as part of the SIOP trial. If this becomes standard treatment there, a future study should look at whether the difference in mortality disappears.

The better prognosis for girls has been noted in other series [Ref 5,9]. Certainly the difference here is striking and perhaps ought to indicate caution in the use of experimental regimes in girls.

The relatively favourable prognosis for those children in Bristol who survived to have radiotherapy, with a 5 year survival of 65%, should be noted. For chemotherapy to further improve survival if it is to be given after radiotherapy than a survival rate at 5 years of greater than 65% seems necessary. In a series of 21 unrandomised patients, a disease free survival of 76% (not actuarial) at a mean duration of 6 years for patients treated with cyclophosphamide and vincristine in addition to surgery and radiotherapy is quoted [Ref 10]. Interestingly enough amongst the 21 patients reported, 1 developed two second malignancies (basal cell carcinoma and parotid carcinoma). Amongst the small number of 8 patients in the SWR who received chemotherapy in addition to radiotherapy 1 (who had received 5500 rads and VM16, D.T.I.C. and Procarbazine) developed a second malignancy (acute leukaemia).

Of the 7 deaths in children before they could have radiotherapy, 4 occurred in children whose tumours were either only partially resectable or unresectable.

Failure to achieve at least a subtotal resection was associated with a high mortality in these patients. Only 1 child out of 3 with partially resected tumour, and none of the 3 children with biopsied only tumour survive.

The existence of more subtle forms of disability after therapy for medulloblastoma will be underestimated by this sort of survey which looks at medical as opposed to educational follow-up. The development of easily administered play performance scales would lead to more accurate evaluation of this area of morbidity [Ref 11]. Chin H. W. et al, found an age dependent effect on learning, particularly in those who had radiation under the age of 4 years. The effects noted in his survey consisted of defects affecting perceptual or visual integration [Ref 12].

### CONCLUSIONS

Brain tumours are the second largest category of childhood malignancy and medulloblastomas make up approximately a quarter of these. Yet in absolute numbers medulloblastomas are rare tumours, approximately 4 presenting each year throughout the South West Region. It is therefore apparent that local studies would need to be continued for considerable time periods so that trends, failures and successes in treatment in any one area could be identified. It would therefore seem sensible for patients to be entered on large national or international trials so that the benefits or disadvantages of the therapies of the next decade can be seen sooner and more clearly. The ability to evaluate therapy in patients with medulloblastoma throughout a region, a country or a continent would be further improved by the adoption of a single staging procedure (such as the MAPS system advocated by Laurent et al) [Ref 13].

The good results for girls in particular, and for both sexes who survive to have radiotherapy of at least 50 Gy, in this series is encouraging progress. This progress has been achieved by radical surgery and by maximising radiotherapy. Over the next decade the main challenge will be to further determine the place of chemotherapy and to improve the quality as well as the quantity of survival.

### REFERENCES

1. American Cancer Society Workshop on Paediatric Brain Tumours, Niagara-on-the-lake, Ontario (1985). *Cancer* **56**, 1743–1904.
2. ELIOT, A. J., SIMPSON, E. M., OAKHILL, A., DECOCK, R. Nystagmus after Medulloblastoma, (submitted for publication).
3. FARWELL, J., DOHRMANN, R., FLANNERY, J. (1984) Oct Medulloblastoma in childhood: an epidemiological study. *J.Neurosurg.* **61(4)**, 657–664.
4. GEROSA, M. A., DI STEFANO, E., OLIVE, A., *et al.* (1981) Multidisciplinary Treatment of Medulloblastoma: a 5-year experience with the SIOP trial. Childs Brains **8**, 107–118.
5. CHATTY, E. M., EARLE, K. M. (1971) Medulloblastoma. A report of 201 cases with emphasis on the relationship of histologic variants to survival. *Cancer* **28**, 977–983.
6. DR BROWN I. H. A survey of 92 children presenting with primary intracranial neoplasmas at Frenchay, Bristol. Unpublished Paper.
7. McINTOSH, N. (1979) Medulloblastoma – a changing prognosis? *Arch.Dis.Child.* **54**, 200–203.
8. STILLER, C. A., LENNOX, E. L. (1983) Childhood medulloblastoma in Britain 1971–77: Analysis of treatment and survival. *Br.J.Cancer.* **48(6)**, 835–41.
9. RAIMONDI, A. J., TOMITA, T. (1979) Medulloblastoma in childhood. Comparative results of partial and total resection. *Child's Brain* **5**, 310–328.
10. McINTOSH, S., CHEN, M., SARTAIN, P. A., DUNCAN, C., FISHBURN, R., KLATSIN, E. H., SCHWARTZ, A. D., VENES, J. L. (1985) Adjuvant Chemotherapy for medulloblastoma. *Cancer.Sept.* **56 (6)**, 1316–1319.
11. LANSKY, L. L., LIST, M. A., LANSKY, S. B., COHEN, M. E., SINKS, L. F. (1985) Towards the development of play performance scale for children. *Cancer* **56**, 1837–1840.
12. CHIN, H. W., YOSH MARUYAMA. (1984) Age at Treatment and long-term results in Medulloblastoma. *Cancer May* **53(9)**, 1952–8.
13. LAURENT, J. P., CHANG, C. H., COHEN, M. E. (1985) A classification system for primitive neuroectodermal tumours (medulloblastoms) of the posterior fossa. *Cancer* **56**, 1807–1809.

## Figures and Tables

**Figure 1 f1:**
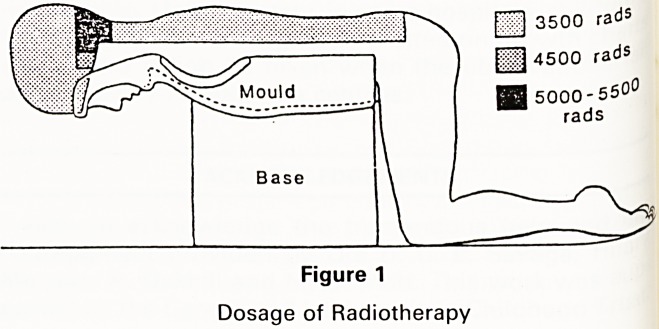


**Figure 2 f2:**
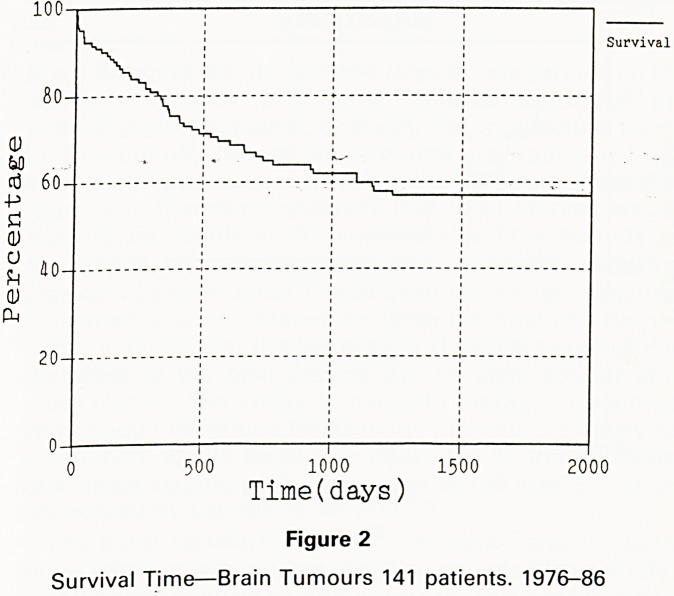


**Figure 3 f3:**
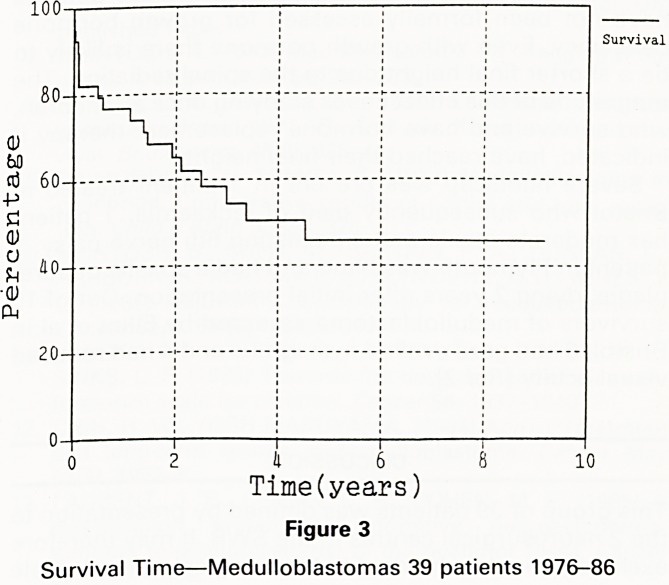


**Figure 4 f4:**
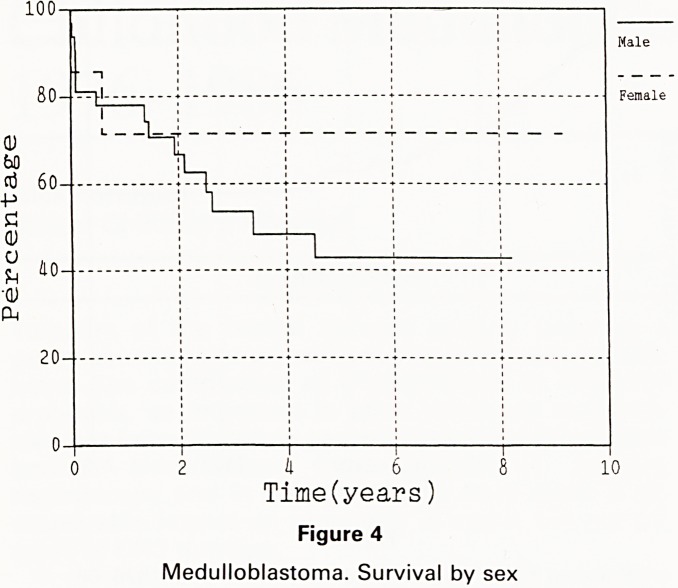


**Figure 5 f5:**
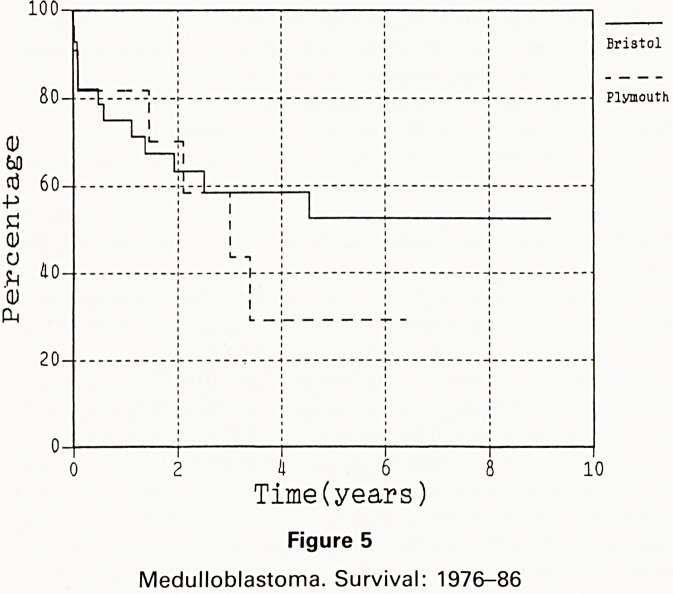


**Figure 6 f6:**
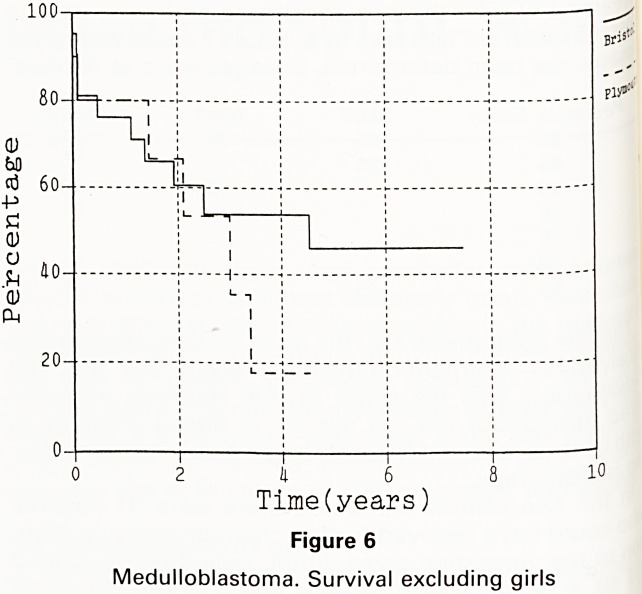


**Figure 7 f7:**